# Injectable Hydrogels Based on Hyaluronic Acid and Gelatin Combined with Salvianolic Acid B and Vascular Endothelial Growth Factor for Treatment of Traumatic Brain Injury in Mice

**DOI:** 10.3390/molecules29081705

**Published:** 2024-04-10

**Authors:** Guoying Zhou, Yajie Cao, Yujia Yan, Haibo Xu, Xiao Zhang, Tingzi Yan, Haitong Wan

**Affiliations:** 1 College of Life Science, Zhejiang Chinese Medical University, Hangzhou 310053, China; gyzhou87@126.com (G.Z.); cyj121123@126.com (Y.C.); yanyujiay@163.com (Y.Y.); kamisdean@163.com (H.X.); 19883206056@163.com (X.Z.); 2 College of Material, Chemistry and Chemical Engineering, Hangzhou Normal University, Hangzhou 311121, China; 3 Institute of Cardio-Cerebrovascular Disease, Zhejiang Chinese Medical University, Hangzhou 310053, China

**Keywords:** traumatic brain injury, vascular endothelial growth factor, salvianolic acid B, bone marrow mesenchymal stem cells, injectable hydrogels

## Abstract

Traumatic brain injury (TBI) leads to structural damage in the brain, and is one of the major causes of disability and death in the world. Herein, we developed a composite injectable hydrogel (HA/Gel) composed of hyaluronic acid (HA) and gelatin (Gel), loaded with vascular endothelial growth factor (VEGF) and salvianolic acid B (SAB) for treatment of TBI. The HA/Gel hydrogels were formed by the coupling of phenol-rich tyramine-modified HA (HA-TA) and tyramine-modified Gel (Gel-TA) catalyzed by horseradish peroxidase (HRP) in the presence of hydrogen peroxide (H_2_O_2_). SEM results showed that HA/Gel hydrogel had a porous structure. Rheological test results showed that the hydrogel possessed appropriate rheological properties, and UV spectrophotometry results showed that the hydrogel exhibited excellent SAB release performance. The results of LIVE/DEAD staining, CCK-8 and Phalloidin/DAPI fluorescence staining showed that the HA/Gel hydrogel possessed good cell biocompatibility. Moreover, the hydrogels loaded with SAB and VEGF (HA/Gel/SAB/VEGF) could effectively promote the proliferation of bone marrow mesenchymal stem cells (BMSCs). In addition, the results of H&E staining, CD31 and α-SMA immunofluorescence staining showed that the HA/Gel/SAB/VEGF hydrogel possessed good in vivo biocompatibility and pro-angiogenic ability. Furthermore, immunohistochemical results showed that the injection of HA/Gel/SAB/VEGF hydrogel to the injury site could effectively reduce the volume of defective tissues in traumatic brain injured mice. Our results suggest that the injection of HA/Gel hydrogel loaded with SAB and VEGF might provide a new approach for therapeutic brain tissue repair after traumatic brain injury.

## 1. Introduction

Traumatic brain injury (TBI) refers to brain damage caused by external mechanical forces, usually resulting in vascular injury, inflammatory activation and brain tissue defects [1]. Secondary injury after mechanical injury can cause cerebral ischemia, hypoxia, neurotoxicity, hematoma and neuroinflammation, which lead to neurodegeneration and apoptosis, resulting in irreversible impediments to nerve regeneration [2,3]. Recent studies have shown that stem cell transplantation and delivery of various neurotrophic factors, growth factors and drugs can improve the recovery of neurological function in TBI [4,5,6]. Transplanted stem cells can differentiate into nerve cells, secrete growth factors, and activate endogenous neurotrophic factors in the appropriate environment [7]. However, the survival rate of the transplanted stem cells is limited due to the unfavorable environment at the injury site of TBI. Growth factors such as vascular endothelial growth factors (VEGFs) have been proven to promote angiogenesis, neuroprotection, and the effects of transplanted stem cells, and thus benefit for brain repair [8]. However, the effects of the factors have been limited by the delivery route, short half-life and long-term release problems [8].

Neural tissue engineering can provide a better delivery route for stem cells, growth factors and drugs, through three-dimensional scaffolds that can mimic the microenvironment in vivo but also allow sustained release of the growth factors and drugs [9,10,11,12,13,14]. Among the different scaffolds, hydrogels are widely used in neural tissue engineering due to their good biocompatibility, physical controllability, and ability to improve the survival rate of stem cells [15,16,17,18,19]. Natural polymer hyaluronic acid (HA) and gelatin have attracted much attention in neural tissue engineering due to their good biocompatibility and biodegradability [20,21,22,23]. Recently, several studies have shown that the combination of HA hydrogel with growth factors and stem cells can effectively improve neurological diseases in animal models by promotion of angiogenesis, neurogenesis, and reduction of neuroinflammation [21,23,24,25]. Gelatin (Gel) is a molecular derivative of collagen that is produced by the irreversible hydrolyzation of the triple helical structure of collagen. It has molecular composition and functional activities similar to those of collagen but is much cheaper, and thus is often used in biomedical applications to replace collagen [26]. Compared with other biopolymer-based hydrogels, Gel-based hydrogels are not only biocompatible, biodegradable, and do not induce antigenicity and toxicity, but also contain important binding moieties that can promote cell attachment and proliferation [27]. Additionally, Gel is rich in reactive residues and is easily gelatinized by chemical crosslinkers such as glutaraldehyde and carbodiimide [28]. Studies have shown that Gel-based hydrogel combined with bone marrow mesenchymal stem cells (BMSCs) can accelerate neurogenesis and tissue defect recovery after brain injury by promoting BMSC differentiation [20,29,30].

In addition, traditional Chinese medicine (TCM) has attracted more and more attention because of its good therapeutic effect on various diseases [31,32,33]. Salvianolic acid B (SAB) has been widely used for neuroprotection and recovery treatment after brain injury due to its anti-inflammatory, anti-oxidation, and angiogenesis effects [34,35,36]. Our previous studies have shown that the combination of SAB in HA hydrogel can promote wound healing via anti-inflammatory and pro-angiogenic effects [37,38,39]. In addition, SAB-loaded hydrogel can effectively improve blood supply and inhibit extracellular matrix degradation after myocardial infarction [40]. The hydrogel loaded with SAB can also be combined with BMSCs to effectively improve intervertebral disc degeneration [41]. Although SAB has been combined with biomaterials to treat a variety of diseases, to the best of our knowledge, there have been no studies using SAB for treatment of brain injury diseases yet.

In this study, we synthesized an HA/Gel hydrogel loaded with SAB and VEGF for treatment of TBI. The gelation time, microstructure, rheological properties, drug release ability, in vitro cytocompatibility and in vivo subcutaneous angiogenesis ability of the hydrogels were investigated. Furthermore, the therapeutic effect of HA/Gel hydrogel loaded with SAB and VEGF on the treatment of TBI was investigated by establishing a C57BL/6 mouse TBI model. The results are reported herein.

## 2. Results

### 2.1. Preparation and Characterization of the Hydrogels

**Figure 1:**
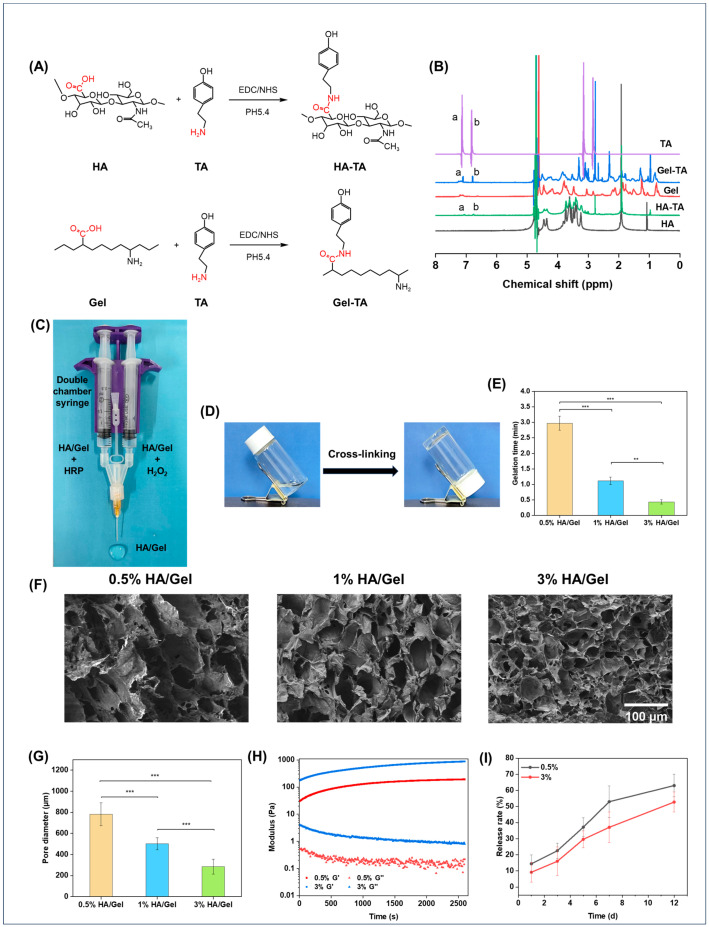
(**A**) Flow chart of the synthesis of HA-TA and Gel-TA. (**B**) ^1^H NMR spectrum of HA, Gel-TA, Gel, HA-TA and TA, a and b represent the peaks at chemical shift 7.1 and 6.8 ppm, respectively. (**C**) The preparation process of the hydrogel. (**D**) The vials were inverted to observe whether the hydrogel had gelled. (**E**) Gelation time diagram of 0.5%, 1%, 3% HA/Gel hydrogels. (**F**) Scanning microscopic morphology of 0.5%, 1%, 3% HA/Gel (Scale bar = 100 μm). (**G**) Pore diameter of 0.5%, 1%, 3% HA/Gel hydrogels. (**H**) Storage modulus (G′) and loss modulus (G″) of 0.5% and 3% HA/Gel hydrogels at different times at 1% strain and 10 rad/s angular frequency. (**I**) Release rate of SAB loaded on 0.5% and 3% HA/Gel hydrogels. Data represent mean ± SD, *n* = 3, ** *p* < 0.01, *** *p* < 0.001.

HA-TA and Gel-TA were synthesized by carbodiimide-mediated coupling of the carboxyl groups in HA to the amino groups in TA, as shown in Figure 1A. The successful synthesis of HA-TA was confirmed by ^1^H NMR. Figure 1B shows the ^1^H NMR spectra of HA, HA-TA, Gel, Gel-TA, and TA. HA-TA and Gel-TA showed new peaks (a and b) at chemical shift (δ) 7.1 and 6.8 ppm compared to HA and Gel, corresponding to the typical amino groups of TA and confirming the successful synthesis of HA-TA and Gel-TA [42,43]. 

The HA/Gel hydrogels were crosslinked through either the C–C linkage between the ortho-carbons of the aromatic ring, or the C–O linkage between the ortho-carbon and the phenolic oxygen [44]. The hydrogels were injected into bottles, and the gelation time was judged by constantly tilting the bottles (Figure 1D). Figure 1E shows the effect of hydrogel concentrations on the gelation time. It was found that gelation times decreased with increasing hydrogel concentrations, ranging from 0.44 to 2.97 min.

The microstructures of the hydrogels were observed by SEM (Figure 1F). Obviously, the hydrogels at all concentrations showed compact porous microstructures. It was also found that the pore size of the hydrogels decreased as the concentration increased. The pore size of the hydrogels at different concentrations was quantitatively summarized, as shown in Figure 1G. The results were consistent with the morphology results, which found that the pore size of the hydrogels was inversely proportional to their concentrations. The quantified values of the pore sizes of 0.5% HA/Gel, 1% HA/Gel, and 3% HA/Gel were 78.18 ± 10.82 μm, 50.29 ± 5.69 μm, and 28.50 ± 6.94 μm, respectively.

The modulus of the hydrogels was investigated by rheometer, as presented in Figure 1H. The results showed that the storage modulus (G′) of the hydrogels increased as the concentration of hydrogels was raised from 0.5% to 3%, which illustrates that the hydrogel cross-linking density increases with increasing concentration. Notably, all hydrogels showed G′ in the range of 100–1000 Pa, which is suitable for brain tissue engineering applications [45]. 

The release profile of SAB from 0.5%, and 3% HA/SAB hydrogels was measured in PBS. As shown in Figure 1I, all hydrogels exhibited controlled and sustainable release of SAB over the examination period. In addition, the higher concentration of HA/Gel/SAB hydrogel at 3% displayed a slower release rate compared to that of the 0.5% concentration. After 12 days, the 0.5% and 3% HA/Gel/SAB hydrogels showed a release rate of 63% and 52%, respectively. 

### 2.2. Cytocompatibility Evaluation of the Hydrogels

**Figure 2:**
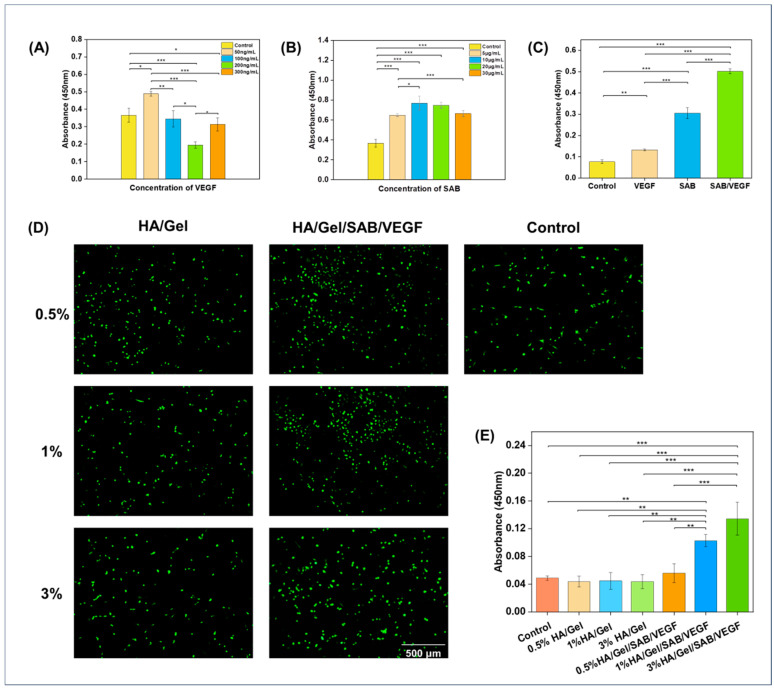
Cytocompatibility evaluation of the hydrogels. (**A**) The optimal concentration of VEGF to promote BMSC proliferation was 50 ng/mL. (**B**) The optimal concentration of SAB to promote BMSC proliferation was 10 μg/mL. (**C**) Effects of the optimal concentration of SAB (10 μg/mL) and VEGF (50 ng/mL) on BMSC proliferation. (**D**) Live/Dead staining figures and (**E**) CCK-8 values of BMSCs after 2 days of incubation with different HA/Gel hydrogel extracts (scale bar = 500 μm). Data represent mean ± SD, *n* = 3, * *p* < 0.05, ** *p* < 0.01 *** *p* < 0.001.

In order to find the optimal concentration of SAB and VEGF uploaded to the hydrogel, their effects on proliferation of BMSCs were detected by CCK-8 kit. As shown in Figure 2A, with the continuous increase in VEGF concentration, the proliferation rate of BMSCs increased firstly and then decreased. The highest proliferation rate was reached when the VEGF concentration was 50 ng/mL. Therefore, the concentration of 50 ng/mL was considered as the optimal VEGF concentration. Similarly, as shown in Figure 2B, the proliferation rate of BMSCs also increased firstly and then decreased as the SAB concentration increased. The proliferation rate reached the highest at the concentration of 10 μg/mL. Therefore, 10 μg/mL was considered as the optimal concentration for SAB. 

To compare the effects of SAB, VEGF and the combination of SAB and VEGF on BMSC proliferation, CCK-8 was applied to detect the proliferation promotion rate of BMSCs. As shown in Figure 2C, the proliferation rate of BMSCs in the SAB/VEGF group was significantly higher than that in the SAB and VEGF groups. The composition of the different hydrogels is shown in Table 1.

To investigate the effects of hydrogel extracts on BMSC proliferation, LIVE/DEAD fluorescent staining and CCK-8 assay were applied. As shown in Figure 2D,E, 0.5% HA/Gel, 1% HA/Gel and 3%HA/Gel had no significant difference compared with the control group. However, the proliferation rates of 0.5% HA/Gel/SABVEGF, 1% HA/Gel/SAB/VEGF and 3% HA/Gel/SAB/VEGF were significantly higher than that of the control group and HA/Gel group. Among them, the 3% HA/Gel/SAB/VEGF had the highest proliferation rate. These results demonstrated not only the excellent cytocompatibility of the HA/Gel hydrogel, but also the promoting effects of HA/Gel/SAB/VEGF hydrogel on BMSC proliferation.

### 2.3. Three-Dimensional (3D) Culture of BMSCs in HA/Gel/SAB/VEGF Hydrogels

**Figure 3:**
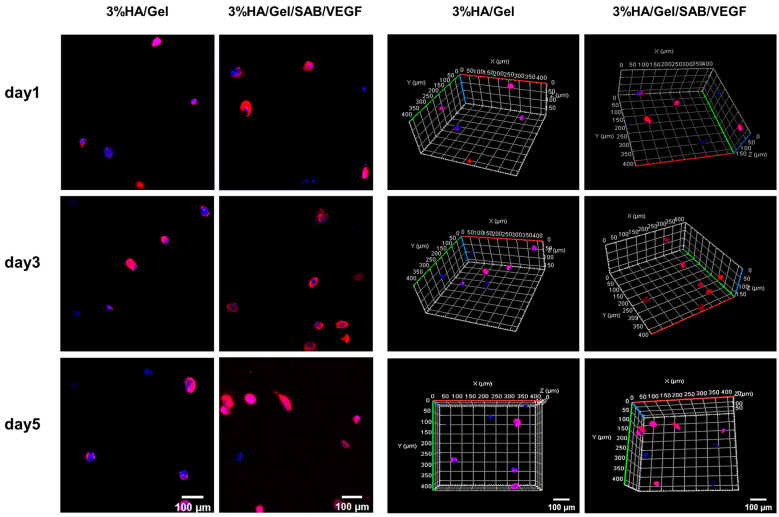
Two-dimensional (2D) (left column, scale bar = 100 μm) and 3D (right column, scale bar = 100 μm) immunofluorescence staining with Phalloidin (red)/DAPI (blue) of BMSCs loaded in 3% HA/Gel/SAB/VEGF hydrogels after 5 days of culture, photographed using confocal microscopy.

Three-dimensional cultures of BMSCs encapsulated in the HA/Gel and HA/Gel/SAB/VEGF hydrogels at days 1, 3 and 5 were stained by phalloidin (red) and DAPI (blue) (Figure 3). The left and right two columns showed the representative 2D and 3D images, respectively. After 5 days of culture, the cells in the blank HA/Gel hydrogel were all round in shape, but a few BMSCs in the HA/Gel/SAB/VEGF hydrogel began to stretch into an oval shape. These results might indicate the potential of the HA/Gel/SAB/VEGF hydrogel in promoting BMSC spreading, as well as the beneficial effects for encapsulation of BMSCs to treat diseases.

### 2.4. Subcutaneous Degradation, Histology and Immunofluorescence Staining

**Figure 4:**
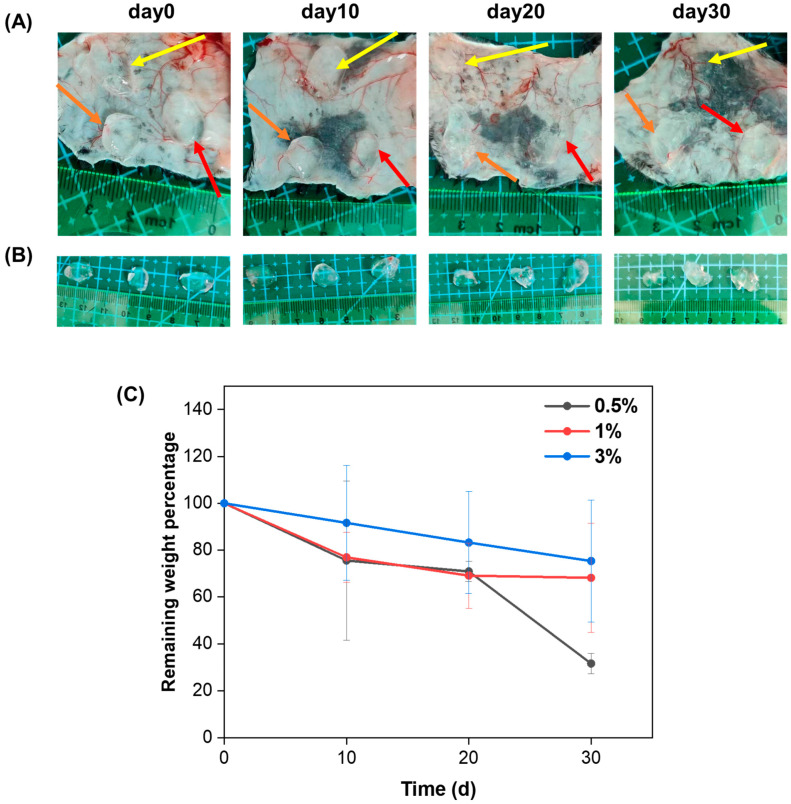
(**A**) Size and morphology of the 0.5% HA/Gel (orange arrows), 1% HA/Gel (red arrows), and 3% HA/Gel (yellow arrows) hydrogels after 10, 20, and 30 days of subcutaneous injection. (**B**) Hydrogels extracted from the subcutaneous tissue of mice at day 0, 10, 20, and 30. From left to right: 0.5%, 1%, and 3%, respectively. (**C**) Comparison of the remaining weight percentages of the hydrogels removed from the subcutaneous tissue of the mice at days 10, 20, and 30. Data represent mean ± SD, *n* = 3.

In order to evaluate the degradation properties of the HA/Gel hydrogels in vivo, 100 μL of the 0.5%, 1%, and 3% HA/Gel hydrogels was injected subcutaneously into the back of the mice, respectively. The subcutaneous hydrogels were extracted and weighed on days 0, 10, 20, and 30. The percentages of remaining weight of the hydrogels were calculated and are shown in Figure 4C. It can be seen that all hydrogels degraded gradually as time progressed. In addition, the degradation rate of the hydrogels decreased as the HA/Gel concentration increased. After 30 days, the remaining weight percentages of 0.5%, 1%, and 3% HA/Gel hydrogels were 31.5%, 68.2%, and 75.3%, respectively. 

**Figure 5:**
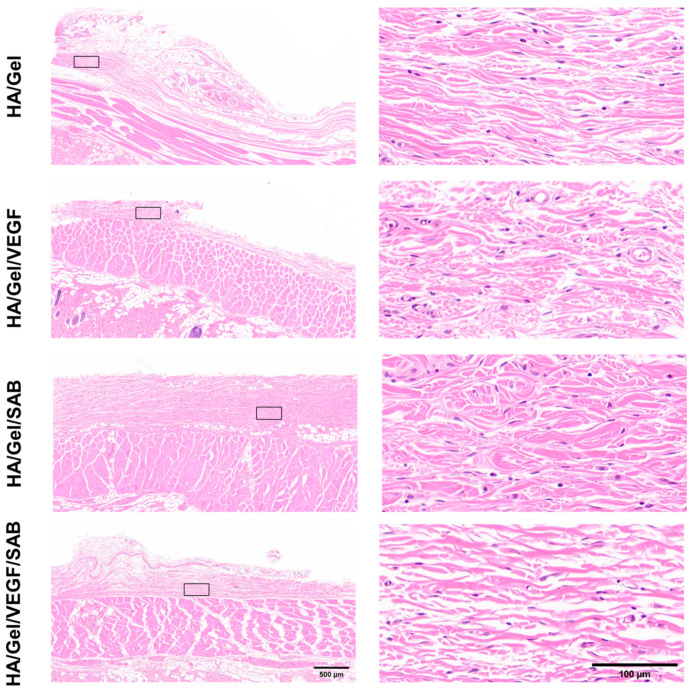
Representative low (**left column**, scale bar = 500 μm) and high (**right column**, scale bar = 100 μm) magnification images of H&E-stained histological sections at 14 days post subcutaneous implantation of different hydrogels.

To further analyze the in vivo compatibility of the hydrogels, H&E staining was performed on the skin tissues around the different hydrogels at day 14 (Figure 5). No obvious inflammatory cells were observed in the skin tissues around all HA/Gel hydrogel samples. The results indicate that the hydrogels possess good compatibility in vivo, which provides support for the injection of hydrogels into the brain tissue of mice for treatment of brain injury.

**Figure 6:**
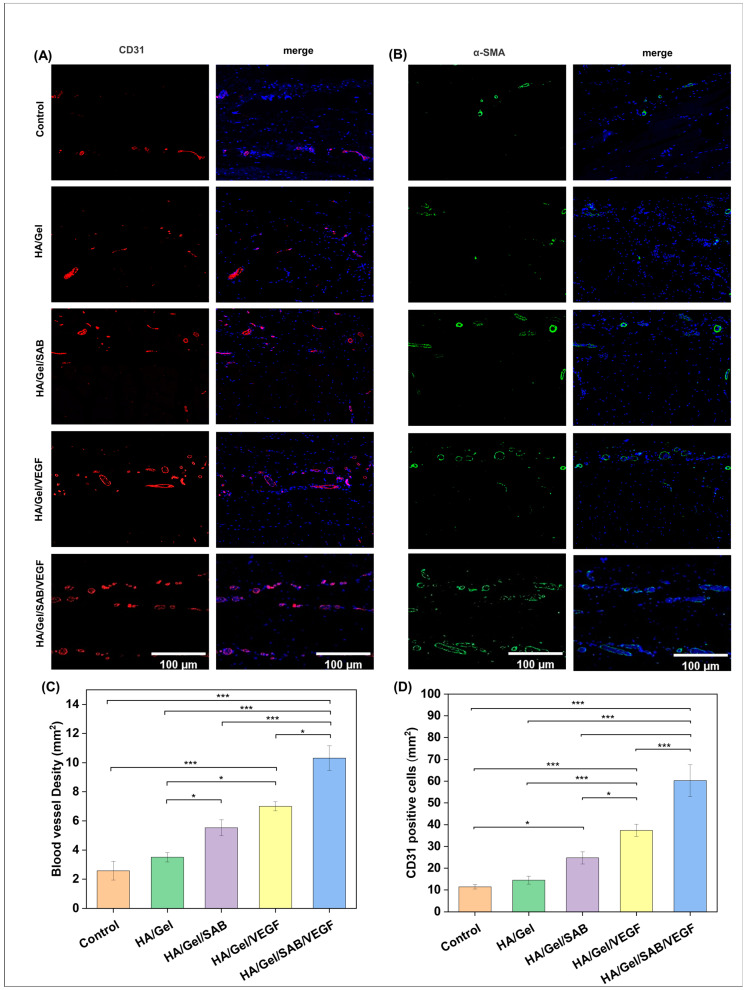
Immunofluorescence staining of (**A**) CD31 (green) and (**B**) α-SMA (red) in skin tissues around different implanted hydrogels on day 14 after subcutaneous injection (Scale bar = 100 μm). (**C**) The quantified number of CD31-positive cells after subcutaneous injection of HA/Gel, HA/Gel/SAB, HA/Gel/VEGF, and HA/Gel/SAB/VEGF hydrogels for 14 days. (**D**) Quantitative graph of blood vessel density on day 14 after subcutaneous injection of HA/Gel, HA/Gel/SAB, HA/Gel/VEGF, and HA/Gel/SAB/VEGF hydrogels. Data represent mean ± SD, *n* = 3, * *p* < 0.05, *** *p* < 0.001.

The angiogenesis-promoting abilities of the hydrogels were evaluated by immunofluorescence staining. As shown in Figure 6A,B, CD31 positive cells (red) and α-SMA positive cells (green) were observed in skin tissues around HA/Gel, HA/Gel/SAB, HA/Gel/VEGF and HA/Gel/SAB/VEGF hydrogels. The quantified data of the mean intensity of CD31-positive cells and quantified blood vessel density are shown in Figure 6C,D. The tissues surrounding HA/Gel/SAB/VEGF hydrogel exerted the highest expression of CD31 and α-SMA compared with other hydrogels. These results indicate that the HA/Gel/SAB/VEGF hydrogel has a significant promoting effect on angiogenesis.

### 2.5. HA/Gel/SAB/VEGF Hydrogel Promotes the Repair of Brain Injury in Mice

**Figure 7:**
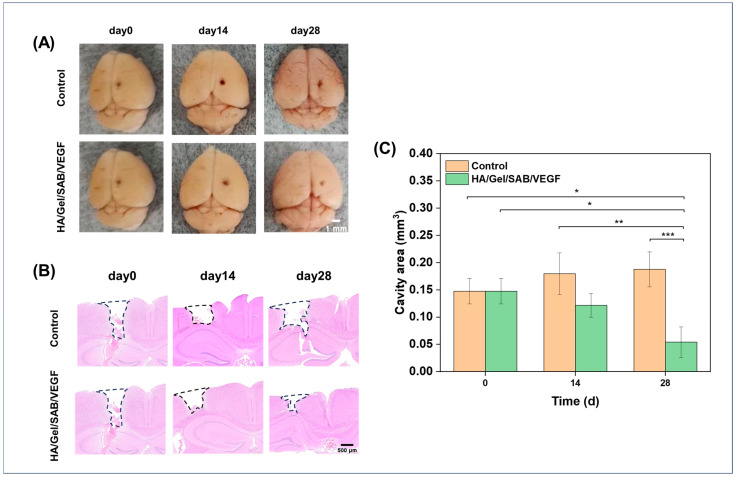
(**A**) Macroscopic observation and (**B**) histological analysis of brain sections with the largest coronal defects, performed by H&E staining after hydrogel implantation at days 0, 14, and 28. The black dashed area indicating the defect area was used for the calculation of defect volume (scale bar = 500 μm). (**C**) Statistical analysis of the volume changes in the injured brain regions of mice on days 0, 14, and 28 after intracerebral injection of the HA/Gel/SAB/VEGF hydrogel, compared with the control group. The volume of the defect was considered as an imaginary cylinder. The section with the largest defect volume in the coronal section was selected, and the defect area from cortex to hippocampus was measured by ImageJ 1.8.0 software. The volume of the defect area was calculated by multiplying the cortical to hippocampal defect area by the diameter measured on the surface of the defect. Data represent mean ± SD, *n* = 3, * *p* < 0.05, ** *p* < 0.01, *** *p* < 0.001.

The effects of HA/Gel/SAB/VEGF hydrogel in repair of brain injury was evaluated by a traumatic brain injury model with cavity formation in mice [46]. After 14 and 28 days of hydrogel injection into the brain defects, macroscopic observations of the brain defects were firstly performed, and the images are shown in Figure 7A. A reduced defect area was found after HA/Gel/SAB/VEGF hydrogel treatment, in comparison to the control group. H&E staining was further applied for a histological analysis of the brain defects. As shown in Figure 7B, the cavity area in the hydrogel-implanted group became smaller, while the area became larger in the control group without any treatment. The quantified value of the cavity area in Figure 7C was consistent with the results in Figure 7B, showing that the HA/Gel/SAB/VEGF hydrogel implantation reduced the defect area and enhanced brain recovery. 

## 3. Discussion

Brain injury results in neuronal death and tissue defects. At present, the methods for treatment of TBI include stem cell transplantation [47,48], injection of growth factors [49,50], traditional Chinese medicine treatment, etc. [51,52,53]. However, the low survival rate and differentiation efficiency after stem cell transplantation, the short half-life of growth factors, and the difficulty of traditional Chinese medicine in crossing the blood–brain barrier limit their therapeutic effects. Tissue engineering strategies can solve these problems by the combination of scaffolds, cells, growth factors and/or drugs. In recent years, injectable hydrogels have been widely used as scaffolds in tissue engineering because of their good biocompatibility, tunable physiochemical properties and controlled drug-release properties.

In this study, an injectable HA/Gel hydrogel was prepared by coupling of the phenol-rich HA-TA and Gel-TA through catalyzation by HRP and H_2_O_2_. The modulus of the HA/Gel hydrogels ranged from 100 to 1000 Pa based on rheological evaluation. According to magnetic resonance elastography (MRE) analysis, the storage modulus of gray matter and white matter in the adult brain is about 3.1 kPa and 2.7 kPa, respectively [54]. The modulus of our HA/Gel hydrogels is close to the strength of the natural brain tissue and thus suitable for brain repair application (Figure 1H). In addition, good biocompatibility is necessary for hydrogels. According to the results of CCK-8 (Figure 2E) and LIVE/DEAD fluorescence staining (Figure 2D), the HA/Gel hydrogel extracts did not affect cell viability. This result could be attributed to the good cytocompatibility of HA and Gel. Hong and others confirmed the HA-TA hydrogel was not toxic to cells [25,55,56]. Ren’s study showed that the gelatin-based hydrogel exhibited good cell compatibility with HUVEC, L929, and 3T3 cells [57]. In addition, bioactive components of TCM and growth factors were shown to promote cell proliferation. Our results showed that SAB and VEGF could promote proliferation of BMSCs (Figure 2A,B), which is consistent with others’ findings [58,59]. Particularly, the combination of SAB and VEGF was found to be much more effective than either SAB or VEGF alone (Figure 2C). Therefore, the SAB and VEGF were both uploaded into the HA/Gel hydrogel to form HA/Gel/SAB/VEGF hydrogel. Sustained and controlled release of the SAB from the hydrogel was observed (Figure 1I). Thereupon, the released SAB and VEGF in the hydrogel extracts exerted proliferation-promoting effects on BMSCs (Figure 2D,E). Moreover, 3D encapsulation experiments showed that the HA/Gel/SAB/VEGF hydrogel possessed the potential to promote the spread and proliferation of BMSCs in 3D condition, indicating the beneficial effects of HA/Gel/SAB/VEGF hydrogel for delivery of BMSCs to treat disease (Figure 3). MSCs can exhibit proliferative behavior in a 3D environment, but this process may take a longer time, perhaps 2 weeks [60], and was not observed in our studies with a period of only 5 days. 

TBI results in a complex inhibitory microenvironment, including ischemia, inflammatory cell proliferation, and formation of glia-derived scar tissue [61,62]. It has been suggested that angiogenesis plays an important role in brain repair [63]. Newly formed blood vessels not only provide oxygen and nutrients to brain tissue but also contribute to neurogenesis and neuronal remodeling [64]. Therefore, promotion of angiogenesis is extremely important for TBI repair. To test the in vivo biocompatibility and the pro-angiogenesis ability, the HA/Gel/SAB/VEGF hydrogel was injected into the subcutaneous tissue of the mice. Firstly, no obvious inflammatory reaction was induced (Figure 5), indicating the excellent biocompatibility in vivo. Furthermore, the HA/Gel/SAB/VEGF hydrogel exhibited excellent angiogenic properties, evidenced by the increased CD31 and α-SMA expression (Figure 6A–D). Finally, the HA/Gel/SAB/VEGF hydrogel was injected into the brain defect site of TBI mice. H&E staining images (Figure 7B) and the quantified data (Figure 7C) showed that the injury volume of the hydrogel group was significantly reduced after 28 days compared with the control group. The favorable repair effects of the HA/Gel/SAB/VEGF hydrogel might be attributed to the spatial support of the hydrogels on the defective cavity and the excellent pro-angiogenic ability of the hydrogels. The underlying mechanism will be studied in the future.

## 4. Materials and Methods

### 4.1. Materials, Cells, and Animals

Sodium hyaluronate (HA, 200–400 k Da), 2-(N-morpholinyl) ethosulfonic acid (MES), 1-(3-Dimethylaminopropyl)-3-ethylcarbodiimide hydrochloride (EDC), N-Hydroxy succinimide (NHS) and Tyramine hydrochloride (TA) were purchased from Aladdin (Shanghai, China). Gelatin (50–100 k Da), was obtained from Sigma (Shanghai, China). Salvianolic acid B (SAB) was purchased from Aifa Biotechnology (Chengdu, China). VEGF-164 was purchased from Cell Signaling Technology (Beverly, MA, USA).

Bone marrow mesenchymal stem cells (BMSCs) were purchased from Fuyuan Biotechnology Co., Ltd. (Shanghai, China), and OriCell^®^ Adult Bone Marrow Mesenchymal Stem Cell Complete Medium was obtained from Cayen (Guangzhou, China). Trypsin and antibiotics (50 units/mL penicillin and 50 units/mL streptomycin) were obtained from Cienry Biotechnology (Huzhou, China). Male C57BL/6 mice (20–25 g) were provided from Shanghai SLAC Laboratory Animal Co., Ltd. (Shanghai, China). The mice were housed in SPF-level animal facilities at 25 °C, with free access to food and water. All of the animal experiments were approved by Zhejiang Chinese Medical University Laboratory Animal Research Center (ZCMULARC).

### 4.2. Synthesis and Characterization of HA-Tyramine (HA-TA) and Gel-Tyramine (Gel-TA)

HA-TA was prepared by formation of a covalent bond between the carboxyl groups in HA and the amino groups in TA. Briefly, HA (1 g, 1% *w*/*v*) was dissolved completely in 100 mL of 2-(N-morpholinyl) ethosulfonic acid (MES) buffer (pH value of around 4.5). Then, 2.5 mmol EDC and 2.5 mmol NHS were added, followed by stirring for 1 h to activate the carboxylate groups in HA. Subsequently, 2.5 mmol TA was added and stirred for 24 h at room temperature. Finally, the reaction solution was transferred to a dialysis bag (MWCO = 3500), dialyzed against NaCl (5 g/L) for 1 day and deionized water for 2 days, and then lyophilized. The freeze-dried product was sealed and stored in the refrigerator at 4 °C. Gelatin (1 g, 1% *w*/*v*) was dissolved completely in 100 mL MES buffer (pH value of around 4.5) at 40 °C., then 25 mmol EDC and 25 mmol NHS were added with stirring for 0.5 h and 1 h, respectively, to activate the Gel carboxylate groups. Afterwards, 25 mmol TA was added and stirred for 24 h at room temperature. Finally, the reaction solution was transferred to a dialysis bag (MWCO = 3500), dialyzed against NaCl (5 g/L) for 1 day and deionized water for 3 days, and then lyophilized. The freeze-dried product was sealed and stored in the refrigerator at 4 °C. The product was characterized by Avance III 600 MHz Digital NMR spectrometer (NMR, 1300623S, Bruker, Ettlingen, Germany).

### 4.3. Hydrogel Fabrication and Physical Characterization

HA-TA and Gel-TA were separately dissolved in PBS to prepare 0.5%, 1%, and 3% (*w*/*v*) solutions. Then, equal concentrations and volumes of HA-TA and Gel-TA were mixed. The final concentrations of HA-TA and Gel-TA in 0.5%, 1% and 3% mixtures were 0.25%, 0.5% and 1.5%, respectively. The mixed solution of HA-TA and Gel-TA, 1.2 U/mL HRP and 3 mM H_2_O_2_ were separately added to a double-inlet syringe to prepare the HA and Gel-based (HA/Gel) hydrogels. The HA/Gel hydrogels of various concentrations (0.5%, 1%, 3% *w*/*v*) were prepared and named as 0.5% HA/Gel, 1% HA/Gel, and 3% HA/Gel. The gelation time varied with the concentration of the hydrogel samples. The SAB-loaded (HA/Gel/SAB) hydrogel, VEGF-loaded hydrogel, and SAB- and VEGF-loaded hydrogel were prepared by adding 1 mg of SAB, 50 ng/mL VEGF,1 mg of SAB and 50 ng/mL VEGF to 1 mL of HA/Gel hydrogel precursor solution individually. Then, the mixtures were mixed with 1.2 U/mL HRP and 3 mM H_2_O_2_ before adding to the double-lumen syringe.

For morphology characterization, the hydrogels were frozen with liquid nitrogen and then freeze-dried. The microstructure of hydrogels with different concentrations was observed by field emission scanning electron microscope (SEM, Hitachi SU8010, Tokyo, Japan) after gold spraying on the fracture surface. Finally, 20 pores were randomly selected for each concentration of the hydrogel samples, and the pore sizes of different hydrogels were compared by ImageJ 1.8.0 software analysis.

The rheological properties of the hydrogels were evaluated by a rheometer (Anton Paar MCR302, Graz, Austria) with PP40 flat plates. The hydrogels were injected between two parallels with a gap of 0.8 mm. The strength testing of the hydrogels was performed at 25 °C at the constant frequency of 10 rad/s and 1% strain to compare the strength of hydrogels of different concentrations.

The release profile of SAB from the HA/Gel/SAB hydrogels was determined in PBS. The HA/Gel/SAB hydrogels (1 mL) were soaked in 3 mL PBS (pH = 7.4) at 37 °C, and 2 mL of the supernatant was collected and replaced with an equal volume of fresh PBS. After all samples were collected, the concentration of SAB in the supernatant was calculated according to a standard curve obtained at an absorbance wavelength of 286 nm in an ultraviolet/visible (UV/Vis) spectrophotometer (Unico-2800, Unico, Franksville, WI, USA). The following equation was used to calculate the percentage of drug release: Cumulative release of SAB (%) = (Released SAB/Total of SAB) × 100%. 

### 4.4. The Cytocompatibility of the HA/Gel Hydrogels

The cytocompatibility of the hydrogels was characterized with bone marrow mesenchymal stem cells (BMSC) by Cell Counting Kit-8 assay (CCK-8, Biosharp, Hefei, China), LIVE/DEAD staining assay (Solarbio, Beijing, China), Phalloidin-iFluor^®^ 555 Conjugate (AAT Bioquest, Inc., Pleasanton, CA, USA) and DAPI Stain (Sigma, Shanghai, China).

For the optimum concentration of SAB and VEGF, BMSCs were seeded with 1 × 10^4^ cells/well in a 24-well plate incubated at 37 °C for 24 h in a 5% CO_2_ incubator. SAB (0 μg/mL, 5 μg/mL, 10 μg/mL, 20 μg/mL) and VEGF (0 ng/mL,50 ng/mL, 100 ng/mL, 200 ng/mL) were added and cultured for another 24 h. Afterwards, the culture medium was removed and washed once with PBS. Then, 100 μL of DMEM containing 10% (*w*/*v*) CCK-8 was added to each well and incubated for 2 h at 37 °C in the dark. The absorbance at 450 nm was measured on a microplate reader (Spectra Max Plus 384, Molecular Devices, Sunnyvale, CA, USA). The wells containing medium without cells served as blank group.

To study the synergetic effects of SAB and VEGF on cell proliferation, BMSCs were seeded with 1 × 10^4^ cells/well in a 24-well plate incubated at 37 °C for 24 h in a 5% CO_2_ incubator. Cells were divided into four groups: control, VEGF (50 ng/mL), SAB (10 μg/mL), and VEGF (50 ng/mL)/SAB (10 μg/mL). After 24 h of incubation, the culture medium was removed, and the cells were washed once with PBS. Then, 100 μL of DMEM containing 10% (*w*/*v*) CCK-8 was added to each well and incubated for 2 h at 37 °C in the dark. The absorbance at 450 nm was measured.

The effect of the hydrogel loaded with SAB and VEGF (HA/Gel/SAB/VEGF) on cell proliferation was assessed by CCK-8 and live/dead staining using hydrogel extracts. The prepared hydrogel precursor solution was filtered through a 0.22 μm sterile filter membrane (Biosharp, Hefei, China). Then, 1 mL of the hydrogel was injected into a 5 mL centrifuge tube. After complete gelation, 3 mL of the complete medium was added to the centrifuge tube and extracted at 37 °C for 24 h. The resulting extract was diluted 10-fold. BMSCs were added to 24-well plates at a concentration of 1 × 10^4^ cells/ well and cultured for 24 h. Afterwards, the old complete medium was discarded, and 1 mL of the diluted hydrogel extracts was added to each well plate. The cells were cultured for another 2 days, and the cell proliferation was measured by CCK-8 kit. LIVE/DEAD staining assay was performed following the manufacturer’s instructions, and 100 μL of staining solution was added per well. After being incubated for 15 min at 37 °C in the dark, the stained cells were visualized using fluorescence microscopy (Leica DMI4000 B, Leica, Wetzlar, Germany). The viable cells were stained with green, and the dead cells were stained with red.

### 4.5. Three-Dimensional (3D) Culture of BMSCs in HA/Gel/SAB/VEGF Hydrogels

For 3D cultures, BMSCs were mixed in 3% HA/Gel/SAB/VEGF hydrogel precursor solutions at a final concentration of 5 × 10^5^ cells/mL, and the concentrations of SAB and VEGF were 1 mg/mL and 50 ng/mL, respectively. A 200 uL volume of the hydrogel was injected into each confocal dish. After complete gelation of the hydrogel, 1 mL of complete medium was added to the dish. The medium was removed on days 1, 3 and 5. After washing, the cells were fixed with 4% paraformaldehyde for 30 min, incubated with 0.1% Triton-X100 at room temperature for 10 min, and sealed with 1% BSA solution at room temperature for 30 min. Afterwards, the cells were incubated with phalloidin dye solution at 37 °C for 1 h in the dark, and then with DAPI working solution at room temperature in the dark for 10 min. After each step, PBS was used for washing 3 times. Finally, the cells were observed and photographed under a confocal microscope (Zeiss, LSM880, Oberkochen, Germany) and analyzed using ZEN 2.3 lite software.

### 4.6. In Vivo Biocompatibility of the HA/Gel Hydrogels

All animal experiments were performed according to the protocols approved by Zhejiang Chinese Medical University, the Ethical Committee, and Laboratory Administration Rules of China. The precursor solutions of HA/Gel hydrogels, HRP (1.2 U/mL) and H_2_O_2_ (3 mM) were filtered through a 0.22 μm sterile filter membrane. Afterwards, 200 μL of the HA/Gel hydrogel precursors was injected into male C57BL/6 mice with a double-inlet syringe. The needles were left for 3 min for gelation of the gels before removal. The mice were euthanized, and the hydrogels were collected to weigh at day 0, 7 and 14.

For histological evaluation, the SAB (1 mg/mL) or/and VEGF (50 ng/mL) were loaded in HA/Gel hydrogel, named as HA/Gel/SAB, HA/Gel/VEGF and HA/Gel/SAB/VEGF hydrogels. Then, 100 μL of each hydrogel was subcutaneously injected into four areas of the back of mice. After the evaluation time points, the mice were euthanized, and the hydrogels were taken out. The surrounding tissues of residual hydrogels were isolated and fixed using 4% paraformaldehyde for hematoxylin-eosin (H&E). 

Immunofluorescence staining of CD31 and α-SMA was used to observe neovascularization in surrounding areas of hydrogels. Briefly, paraffin sections were deparaffinized to water and blocked by addition of 3% BSA (G5001, Servicebio, Wuhan, China) for 30 min after antigen repair. After removing the blocking solution, rabbit clone antibody of CD31 (1:200, GB11063-2, Servicebio, Wuhan, China) or α-SMA (1:400, GB111364, Servicebio, Wuhan, China) was added and incubated overnight at 4 °C. After washing, the corresponding secondary antibody Cy3-labeled goat anti-rabbit IgG (1:300, GB21303, Servicebio, Wuhan, China) or Alexa Fluor488-labeled goat anti-rabbit IgG (1:400, GB25303, Servicebio, Wuhan, China) was added and incubated at room temperature for 50 min. The cells were counterstained with DAPI (G1012, Servicebio, Wuhan, China) for 10 min at room temperature, followed by the addition of autofluorescence quench agent (G1221, Servicebio, Wuhan, China) for 5 min. The slides were sealed and examined under a fluorescence microscope (Nikon Eclipse C1, Nikon, Tokyo, Japan) and quantitatively analyzed by ImageJ.

### 4.7. Establishment of TBI Model and Hydrogel Implantation

A TBI model was established by using 7- to 8-week-old C57BL/6 mice [46]. After anesthesia with isoflurane, the mice were immobilized on a brain stereotaxic device. After exposure of skull and removal of the periosteum, a cranial window of 3 mm in diameter was drilled with a skull drill after alcohol wiping. Then, the meninges were removed, and the cerebral cortex was exposed. Afterwards, the brain parenchyma was aspirated with a flat head needle (approximately 1 mm in diameter) to create a cylindrical tissue defect of around 1 mm in diameter and 1 mm in depth. This method may cause bleeding. After controlling bleeding with sterile cotton swabs and saline, HA/Gel and HA/Gel/SAB/VEGF hydrogels were injected into the defect areas. The cranial window was then covered with sterile bone wax after the hydrogel was completely solidified. Finally, the scalps of the mice were sutured, and each animal was placed on a heating pad to restore body temperature until returning to movement. The mice were given normal feeding after surgery. Brains were harvested on days 0, 14, and 28 for H&E staining analysis.

### 4.8. Statistical Analysis

ImageJ 1.8.0 and Origin 9.0 software were used for analysis and mapping. All experiments were repeated three times, and the results are expressed as the mean ± standard error (SD). Analysis of variance (ANOVA) was used to analyze the experimental data. * *p* < 0.05, ** *p* < 0.01 and *** *p* < 0.001, a value of *n* = 3 was considered statistically significant.

## 5. Conclusions

In this study, we developed an injectable HA/Gel hydrogel based on HA and gelatin, and loaded with VEGF and SAB. The HA/Gel hydrogels showed porous structures, suitable rheological properties, and sustained release of SAB. In vitro 2D cell experiments demonstrated the good cell compatibility and the proliferation-promoting abilities of the HA/Gel/SAB/VEGF hydrogel. Three-dimensional cell experiments indicated that the internal microenvironment of the HA/Gel/SAB/VEGF hydrogel is suitable for BMSC growth. Furthermore, the HA/Gel/SAB/VEGF hydrogel significantly promoted the expression of CD31 and α-SMA, indicative of good angiogenesis effects. Moreover, the injection of the HA/Gel/SAB/VEGF hydrogel significantly reduced the defective volume and promoted brain recovery compared with the control group. Overall, our results suggest that the fabricated HA/Gel/SAB/VEGF hydrogel could provide a promising strategy for the treatment of traumatic brain injury.

## Figures and Tables

**Table 1:** The composition of the different hydrogels (1 mL).

Sample	HA-TA (mL)	Gel-TA (mL)	SAB (mg)	VEGF (ng)
HA/Gel	0.5	0.5	0	0
HA/Gel/SAB	0.5	0.5	1	0
HA/Gel/VEGF	0.5	0.5	0	50
HA/Gel/SAB/VEGF	0.5	0.5	1	50

## Data Availability

The data that support the findings of this study are available on request from the corresponding author.
